# A Humanized Anti-CD22-Onconase Antibody-Drug Conjugate Mediates Highly Potent Destruction of Targeted Tumor Cells

**DOI:** 10.1155/2015/561814

**Published:** 2015-10-28

**Authors:** Tobias Weber, Athanasios Mavratzas, Stefan Kiesgen, Stephanie Haase, Benedikt Bötticher, Evelyn Exner, Walter Mier, Ludger Grosse-Hovest, Dirk Jäger, Michaela A. E. Arndt, Jürgen Krauss

**Affiliations:** ^1^Department of Medical Oncology, National Center for Tumor Diseases, Heidelberg University Hospital, 69120 Heidelberg, Germany; ^2^Department of Nuclear Medicine, Heidelberg University Hospital, 69120 Heidelberg, Germany; ^3^Department of Immunology, University of Tübingen, 72076 Tübingen, Germany; ^4^Immunotherapy Program, National Center for Tumor Diseases, German Cancer Research Center (DKFZ), 69120 Heidelberg, Germany

## Abstract

Antibody-drug conjugates (ADCs) have evolved as a new class of potent cancer therapeutics. We here report on the development of ADCs with specificity for the B-cell lineage specific (surface) antigen CD22 being expressed in the majority of hematological malignancies. As targeting moiety a previously generated humanized anti-CD22 single-chain variable fragment (scFv) derivative from the monoclonal antibody RFB4 was reengineered into a humanized IgG1 antibody format (huRFB4). Onconase (ranpirnase), a clinically active pancreatic-type ribonuclease, was employed as cytotoxic payload moiety. Chemical conjugation via thiol-cleavable disulfide linkage retained full enzymatic activity and full binding affinity of the ADC. Development of sophisticated purification procedures using size exclusion and ion exchange chromatography allowed the separation of immunoconjugate species with stoichiometrically defined number of Onconase cargos. A minimum of two Onconase molecules per IgG was required for achieving significant *in vitro* cytotoxicity towards lymphoma and leukemia cell lines. Antibody-drug conjugates with an Onconase to antibody ratio of 3 : 1 exhibited an IC_50_ of 0.08 nM, corresponding to more than 18,400-fold increased cytotoxicity of the ADC when compared with unconjugated Onconase. These results justify further development of this ADC as a promising first-in-class compound for the treatment of CD22-positive malignancies.

## 1. Introduction

The incidence of B-cell neoplasms in Europe has been estimated at approximately 21 per 100,000 [1]. Modern treatment concepts increasingly take phenotype, genotype, and risk factors into consideration. Optimization of conventional cytostatic regimens through addition of tumor-specific anti-CD20 monoclonal antibodies (mAbs) or dose intensification followed by autologous/allogeneic stem cell transplantation has significantly improved treatment outcome of B-cell neoplasms over the last years [2].

However, many patients eventually succumb either to treatment-refractory disease or to severe treatment-related side effects [3, 4]. This necessitates the development of target-directed anticancer therapies with increased antitumor efficacy, yet acceptable systemic toxicity. Antibody-drug conjugates (ADCs) harness the targeting function of monoclonal antibodies towards tumor-associated antigens (TAA) to deliver potent cytotoxic drugs. ADCs have progressed to phase III trials and the first such compounds approved were gemtuzumab ozogamicin and brentuximab vedotin for the treatment of acute myeloid leukemia and relapsed Hodgkin and anaplastic large cell lymphoma, respectively. With only modest complete remission rates of 30% [5] and unexpectedly severe postapproval toxicity that in part outweighed its clinical benefit [6] gemtuzumab ozogamicin has been withdrawn in the US in 2010. More recently trastuzumab emtansine (T-DM1) has been approved for the treatment of metastasized HER2-positive breast cancer [7]. For the treatment of hematologic malignancies several other ADCs, targeting CD79b, CD74, CD33, CD30, CD22, and CD19, are currently in clinical development. Prerequisite for the antitumoral activity of ADCs is sufficient cellular internalization of the compound upon TAA-binding, followed by the intracellular release of the carried payload [8]. The B-cell lineage restricted receptor CD22, being overexpressed in the majority of B-cell non-Hodgkin lymphomas (B-NHL) [9], as well as in B-cell precursor acute lymphoblastic leukemia (BCP-ALL) [10], is a particularly attractive target for ADC approaches. This is due to the very rapid and sustained internalization of the targeted receptor [11, 12] and its absence on hematopoietic stem cells [13]. Inotuzumab ozogamicin (CMC-544), an anti-CD22-calicheamicin ADC, has been extensively studied in patients with both indolent and aggressive B-cell NHL as well as acute leukemias [14]. Several phase I and II studies conducted with inotuzumab ozogamicin demonstrated in part highly significant clinical activity across all explored entities. However, in 2013 an ongoing phase III study in patients with aggressive B-NHL was discontinued since an interim analysis of overall survival demonstrated no statistically significant superiority of CMC-544 in combination with rituximab over the comparator arm. The press release reporting on the study termination concluded that “hematologic cancers are a complex group of diseases, with more than 70 different types of lymphomas, leukemias or myelomas that require unique treatment options.” Therefore, clinical development of anti-CD22 ADCs with alternative payloads remains of utmost importance. The murine anti-CD22 IgG1 mAb RFB4 and a disulfide antibody fragment derivative, dsFv-RFB4, have been covalently linked to plant toxins or genetically fused to bacterial toxins, respectively [15–19]. From these compounds particularly the recombinant immunotoxin BL22 has produced highly impressive clinical results [20]. However, administration of BL22 was associated with severe adverse effects such as immunogenic reactions and in a few cases development of capillary leak syndrome. As a consequence, a higher affinity antibody fragment derivative for linkage to the bacterial toxin has been developed and the compound (HA22, CAT 8015) exhibited a more favorable toxicity profile, yet similar potent activity as its predecessor in a phase I trial in patients with chemotherapy-resistant hairy cell leukemia [21].

Valuable payload alternatives to bacterial toxins are ribonucleases from the pancreatic ribonuclease (RNase) A superfamily with near absence of immunogenicity [22]. Onconase (ONC, ranpirnase), a 12 kDa basic single-chain protein, originally isolated from oocytes of* Rana pipiens* [23], kills tumor cells with an LD_50_ of 10^−7 ^M which is comparable to the potency of maytansinoids and auristatins. The antitumor effects of ONC can be ascribed to tRNA- [24, 25], dsRNA- [26], and miRNA-cleavage [26, 27], as well as to transcriptional gene regulation interactions [28]. In phase I/II clinical trials for treatment of various solid tumors and malignant mesothelioma ONC was immunologically well tolerated and displayed acceptable and reversible systemic toxicity [29, 30]. After conjugation to the murine anti-CD22 IgG2a-mAb LL2 [31], ONC caused only mild off-target toxicity in lymphoma xenografted mice, and the toxic total cumulative dose (TCD) was reached only at concentrations of >300 mg/kg body weight. In comparison, a* Pseudomonas* exotoxin-LL2 immunoconjugate caused 100% lethality in mice at a TCD of 7 mg/kg [32]. Thus, ONC seems to present a promising payload for anti-CD22 immunoconjugates by combining high antitumor potency and low systemic toxicity.

In this study we report on the generation, purification, and* in vitro *characterization of different ONC-based anti-CD22 immunoconjugates. As antibody targeting moiety we reengineered the previously humanized RFB4 scFv [33] into a humanized IgG1. We tested two different chemical conjugation strategies using various crosslinkers for noncleavable and thiol-cleavable linkage and different ONC payload formats. Sophisticated purification procedures were used for obtaining active ADCs with distinct molar drug to antibody ratios (DARs). We show that immuno-RNases were able to kill targeted lymphoma and leukemia cells in a DAR-dependent manner.

## 2. Materials and Methods

### 2.1. Materials

All tumor cell lines were purchased from ATCC (Manassas, VA, USA). Onconase (ranpirnase) was a kind gift from Kuslima Shogen (Alfacell Corporation, New Jersey, USA).

*AhdI* and* Sfi*I were obtained from New England Biolabs (Ipswich, USA). The RNase substrate poly-rU was acquired from Amersham Biosciences (Little Chalfont, UK); the fluorogenic substrate 6-FAM-dArUdGdA-BHQ-1 (6-Carboxyfluorescein-dArUdGdA-Black-Hole-Quencher-1) was from biomers.net (Ulm, Germany). FITC- (fluorescein isothiocyanate-) conjugated secondary antibodies were obtained from Jackson ImmunoResearch (Laboratories, West Grove, PA, USA); the control antibody RFB4 was from Santa Cruz Biotechnology (Dallas, Texas, USA). BenchMark Prestained Protein Ladder, DMEM (Dulbecco's Modified Eagle's Medium), 2-mercaptoethanol, NuPAGE LDS Sample Buffer (4x), Novex HIMark Pre-Stained Protein Standard, Novex Tris-Acetate SDS Running Buffer, and Novex NuPAGE 3–8% Tris-Acetate Gels were purchased from Life Technologies GmbH (Darmstadt, Germany). RunBlue LDS Sample Buffer, RunBlue Rapid SDS Run Buffer, and RunBlue 12% SDS Gels were acquired from Expedeon (Harston, UK). FBS (fetal bovine serum), PBS (phosphate buffered saline), penicillin, RPMI-1640 (Roswell Park Memorial Institute) medium, sodium azide, and streptomycin were obtained from Sigma-Aldrich (St. Louis, MO, USA). AlamarBlue, 2-IT (2-iminothiolane), SMCC (succinimidyl 4-(*N*-maleimidomethyl) cyclohexane-1-carboxylate), SPDP (*N*-succinimidyl 3-(2-pyridyldithio) propionate), and Spectra Multicolor Broad Range Protein Ladder were procured from Thermo Fisher Scientific (Waltham, MA, USA). APS (ammonium persulfate), coomassie brilliant blue R-250, DMSO (dimethyl sulfoxide), DTT (dithiothreitol), G418 (geneticin disulfate), glycerol, isopropanol, MES (2-(N-morpholino) ethanesulfonic acid), NaCl (sodium chloride), Rotiphorese Gel 30 acrylamide/bisacrylamide solution, SDS (sodium dodecyl sulfate), toluidine blue, Tris-HCl (Tris(hydroxymethyl)aminomethane hydrochloride), and ZelluTrans/Roth dialysis membranes were provided by Carl Roth GmbH (Karlsruhe, Germany). CDM HD (Chemically Defined Medium High Density) serum replacement and the hollow fiber cell culture bioreactor were purchased from FiberCell Systems (Frederick, USA). Amicon Ultra centrifugal filter units, EDTA (ethylenediaminetetraacetic acid, disodium salt dihydrate), and Millex-GV sterile filter units were purchased from Merck KGaA (Darmstadt, Germany). TEMED (N,N,N,N-tetramethylethylenediamine) was obtained from Fluka Biochemika (Buchs, Switzerland). Cell culture plates were purchased from Greiner Bio-One (Kremsmünster, Austria). All columns used for purification were provided by GE Healthcare (Little Chalfont, UK).

### 2.2. Generation, Production, and Purification of Humanized IgG huRFB4

For generation of a humanized anti-CD22 IgG1, variable domain genes of the previously humanized scFv SGIII [33] were synthesized (Entelechon GmbH, Bad Abbach, Germany) with splice donor and acceptor signal sequences and cloned into eukaryotic expression vectors containing regulatory elements of the immunoglobulin locus, a human constant heavy *γ*1 chain, and a human constant *κ* chain, respectively. Heavy chain and light chain plasmids of the humanized antibody construct were linearized with* AhdI* and* Sfi*I, respectively, and transfected into Sp2/0-Ag14 mouse myeloma cells by electroporation (230 V, 975 *μ*F). Cells were grown by limiting dilution in selective media (DMEM, 10% FBS, 50 *μ*M 2-mercaptoethanol, and 1 mg/mL G418) for 2-3 weeks to obtain single-cell clones. Positive clones were identified by flow cytometric analysis and monitored for IgG secretion rates by Dot Blot using a purified mAb as reference standard. The highest producing clone for huRFB4 IgG was expanded to T-175 flasks, adapted to high glucose DMEM culture media supplemented with 10% CDM HD serum replacement, and subsequently inoculated into a hollow fiber cell culture bioreactor. IgGs were purified from cell culture supernatants by protein A chromatography using a HiTrap rProtein A FF column and dialyzed against PBS (pH 7.4). Purity was assessed by analytical size exclusion chromatography on a Superdex 200 10/300 GL column as ≥95%.

### 2.3. Chemical Conjugation

Intermolecular protein conjugation of ONC and mAb huRFB4 was performed using as heterobifunctional cross-linking agents either succinimidyl 4-(*N*-maleimidomethyl) cyclohexane-1-carboxylate (SMCC) or* N*-succinimidyl 3-(2-pyridyldithio) propionate (SPDP) for noncleavable and thiol-cleavable linkage, respectively. SMCC and SPDP solutions were freshly prepared in DMSO and diluted 1 : 10 in PBS (pH 7.4) shortly before use. To generate SMCC-based immunoconjugates purified huRFB4 IgG antibody (1 mg/mL) was incubated with 40-fold molar excess of SMCC in PBS (pH 7.4) containing 5 mM EDTA for 30 min at room temperature. ONC (2 mg/mL) was simultaneously reduced to des(30-75)-ONC in PBS (pH 7.2) in the presence of 5 mM DTT and 0.2 nM EDTA for 120 min at 15°C [34]. SMCC and DTT were removed by dialysis against PBS (pH 7.4) containing 5 mM EDTA. The maleimide activated antibody was added to a 4.0-fold molar excess of des(30-75)-ONC and the reaction mixture was incubated for 30 min at room temperature.

In order to prepare SPDP-based immunoconjugates, purified huRFB4 IgG antibody (1 mg/mL) was incubated with a 40-fold molar excess of 2-iminothiolane (2-IT) in PBS (pH 8.0) in the presence of 5 mM EDTA for 60 min at room temperature. ONC (2 mg/mL) was simultaneously incubated with a 2.0-fold molar excess of SPDP in PBS (pH 7.4) in the presence of 5 mM EDTA for 60 min at room temperature. Excess reagents were removed using PD-MiniTrap G-25 columns equilibrated with PBS (pH 7.4) containing 5 mM EDTA. Both modified proteins were combined and incubated overnight at 4°C for conjugation using a 10-fold molar excess of pyridyldithiol-activated ONC.

### 2.4. Purification of ADCs with Distinct Molar Drug to Antibody Ratio

All chromatographic runs were performed at room temperature. The SMCC-based immunoconjugate preparations were subjected to preparative size exclusion chromatography (SEC) on a Superdex 200 10/300 GL column equilibrated and eluted with PBS (pH 7.4) at a flow rate of 0.5 mL/min. Preparative SEC of the SPDP-based immunoconjugate preparations was performed on a HiLoad 16/60 Superdex 200 pg column with a flow rate of 0.3 mL/min using 20 mM MES, 50 mM NaCl at pH 6.0 as elution buffer. Antibody-ONC conjugate populations isolated by SEC were subjected to preparative ion exchange chromatography (IEX) on a Mono-S 5/50 GL column at a flow rate of 1 mL/min. SPDP immunoconjugates with different ONC-mAb ratios were separated using 20 mM MES (pH 6.0) with a linear NaCl gradient (50 mM–1 M) as elution buffer. Specific IEX peak fractions were collected and dialyzed against PBS (pH 7.4). Purified protein samples were concentrated using centrifugal filter units, sterile-filtered, and stored at 4°C. Purity and identity of concentrated immunoconjugates were analyzed by calibrated analytical SEC using a Superdex 200 10/300 GL column.

### 2.5. Determination of Protein Concentration

Concentrations of homogenously purified proteins were calculated from the absorbance at 280 nm measured on a NanoDrop ND-1000 spectrophotometer (Thermo Fisher Scientific, Waltham, MA, USA) using the Beer-Lambert law with the molar absorption coefficient *ε* and the molecular weight calculated for each individual compound. The calculated *ε* value for the unmodified IgG was 203,900 M^−1^·cm^−1^, for ONC 10,010 M^−1^·cm^−1^, and for the chemically linked immuno-RNases carrying one, two, or three ONC payloads 213,910 M^−1^·cm^−1^, 223,920 M^−1^·cm^−1^, and 233,930 M^−1^·cm^−1^, respectively. The molecular weight was set at 150,000 g/mol for the unmodified IgG, 11,840 g/mol for ONC, and 161,840 g/mol (OAR 1 : 1), 173,680 g/mol (OAR 2 : 1), and 185,520 g/mol (OAR 3 : 1) for the immunoconjugates. The extinction coefficient and the molecular weight for immunoconjugates with determined sizes of 250–290 kDa were calculated for an ADC with an average payload of 10 RNases and set at 304,000 M^−1^·cm^−1^ and 268,400 g/mol, respectively. To compare antigen binding activity, cytotoxicity, and ribonucleolytic activity of generated immunoconjugates, IgG and ONC determined protein concentrations were converted to molarity.

### 2.6. SDS-PAGE and Densitometry

Protein samples were analyzed by sodium dodecyl sulfate polyacrylamide gel electrophoresis (SDS-PAGE) performed under nonreducing conditions on precast 3–8% Tris-Acetate gels using Tris-Acetate buffer and under reducing conditions on precast 12% Bis-Tris gels using Tris-MOPS buffer followed by detection by staining with coomassie brilliant blue. Novex HIMark Pre-Stained Protein Standard and Spectra Multicolor Broad Range Protein Ladder were used as protein standards. Coomassie stained protein bands on reducing SDS-PAGE gels of SPDP-based ADCs were captured on an Epson Perfection V750 Pro scanner (SEIKO Epson Corporation, Nagano, Japan) and analyzed by Photoshop Elements 10 software (Adobe Systems Incorporated, San Jose, USA). After converting to greyscale and applying the invert filter, mean densities of ONC protein bands were determined by histogram analyses.

### 2.7. Cell Binding Analysis

Specific binding of huRFB4 IgG was determined by flow cytometry using the human CD22-positive B-cell lines Daudi, Raji, and Ramos and the human CD22-negative T-cell line Jurkat. Bound huRFB4 IgG and chemically linked immuno-RNases were detected using a FITC-conjugated rabbit antibody specific for the Fc region of human IgG. The mouse monoclonal control antibody RFB4 was detected by staining with an FITC-conjugated goat anti-mouse IgG. Fluorescence recordings were made on a FACSCanto II flow cytometer (BD Biosciences, San Jose, CA, USA) and median fluorescence intensity (MFI) was calculated using the FACSDiva software (BD Biosciences, San Jose, CA, USA). Background fluorescence was determined using cells incubated with FITC-conjugated secondary antibody under the same conditions. Equilibrium-binding curves were determined by incubating 5 × 10^5^ Raji cells in triplicate with serial dilutions of either murine RFB4, huRFB4 IgG, or chemically linked immuno-RNases for 2 h at room temperature in 100 *μ*L PBS-FACS buffer containing 2% FBS and 0.1% sodium azide. After two washes with 200 *μ*L FACS buffer bound antibodies were detected as described above. Background fluorescence was subtracted from measured median fluorescence and relative affinities were determined according to the Levenberg-Marquardt algorithm for nonlinear regression using GraphPad Prism 5.0 (GraphPad Software, La Jolla, CA, USA).

### 2.8. Analysis of Ribonucleolytic Activity

Ribonucleolytic activity of SMCC-based immunoconjugates was investigated by zymogram gel electrophoresis as previously described [35]. Briefly, samples were prepared in zymogram loading buffer containing final concentrations of 62.5 mM Tris-HCl, pH 6.8, 10% glycerol, and 2.5% SDS and separated under nonreducing conditions on 12% polyacrylamide gels containing 0.3 mg/mL poly-rU in the separating gel as a substrate for ONC. After electrophoresis the gel was washed twice using 10 mM Tris-HCl, pH 8.0, containing 20% isopropanol to remove SDS, and placed in renaturating buffer containing 100 mM Tris-HCl, pH 8.0 for 12 h (4°C). RNase activity was detected by staining with 0.2% (w/v) toluidine blue in 10 mM Tris-HCl, pH 8.0 for 10 min, followed by destaining with 10 mM Tris-HCl, pH 8.0, until visualization of the ribonucleolytic activity bands was obtained. BenchMark Prestained Protein was used as protein standard.

Ribonucleolytic activity of SPDP-based immuno-RNases and ONC was quantified using the fluorogenic substrate 6-Carboxyfluorescein-dArUdGdA-Black-Hole-Quencher-1 (6-FAM-dArUdGdA-BHQ-1). Increase in fluorescence after cleavage of substrate was monitored over time using an Infinite F200 Pro microplate reader (Tecan Group, Männedorf, Switzerland) with a 485/535 nm (excitation/emission) filter set. Reactions were carried out in black 96-well plates in 100 mM MES-NaOH buffer (pH 6.0) containing 100 mM NaCl and 6-FAM-dArUdGdA-BHQ-1 (5 nM) at (25 ± 2)°C in a total reaction volume of 200 *μ*L per well. Buffer served as negative control and an excess concentration of RNase A was used as positive control. Values of *k*_cat_/*K*_*M*_ were calculated using

(1) $$\begin{array}{c} \frac{k_{\text{cat}}}{K_{M}}=\frac{\left(\Delta F/\Delta t\right)}{\left(F_{\max}-F_{0}\right)\cdot \left[E\right]}, \end{array}$$

where Δ*F*/Δ*t* represents the initial reaction velocity, *F*_0_ the initial fluorescence intensity before addition of RNase, *F*_max⁡_ the fluorescence intensity after complete cleavage of the substrate by excess RNase A, and [*E*] the RNase concentration. At least three independent assays were performed.

### 2.9. Cytotoxicity Assay

Human lymphoma and leukemia cells were maintained in RPMI 1640 supplemented with 10% fetal bovine serum, 100 U/mL penicillin, and 100 *μ*g/mL streptomycin. Daudi were seeded at a density of 2 × 10^4^/100 *μ*L, while Nalm6 and Jurkat were seeded at a density of 1 × 10^4^/100 *μ*L into 96-well flat-bottom plates and incubated with various concentrations of protein or buffer as control at 37°C, 5% CO_2_ for 72 h in a total volume of 110 *μ*L. In order to determine the viability, cells were incubated with 10 *μ*L alamarBlue per well at 37°C, 5% CO_2_ for 4 h. Absorbance was measured at 570 nm (reference: 620 nm) using an Infinite F200 Pro microplate reader (Tecan Group, Männedorf, Switzerland). Cell viability was expressed as percentage of viable cells treated with protein related to buffer control. The concentration required to inhibit cell viability by 50% relative to buffer-treated control cells was defined as IC_50_ (half maximal inhibitory concentration) and was determined from semilogarithmic plots. At least two independent assays with each assay containing triplicates were performed.

## 3. Results

### 3.1. Reformatting of Humanized Anti-CD22 huRFB4 scFv into Humanized Anti-CD22 huRFB4 IgG for Subsequent Generation of Chemical Immunoconjugates

**Figure 1:**
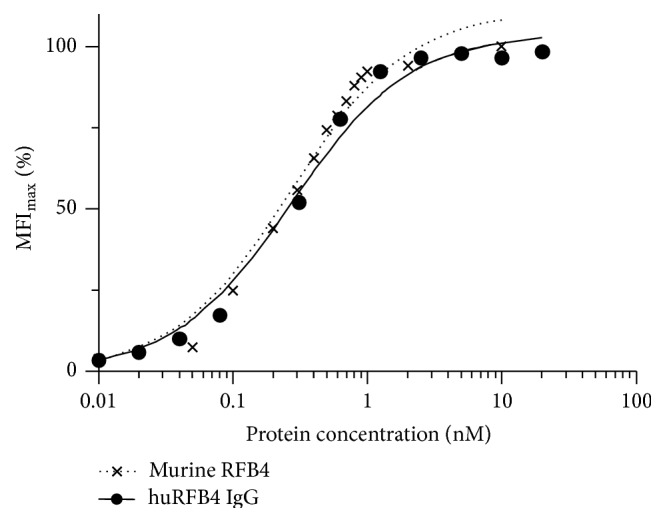
Equilibrium-binding curves of murine RFB4 and huRFB4 IgG. Binding activity to CD22-positive Raji cells at indicated concentrations was determined by flow cytometry performing triplicate measurements. Bars represent standard errors (SEs) of mean values. MFI_max_: maximum median fluorescence intensity.

We have previously grafted the specificity of the murine anti-CD22 monoclonal antibody (mAb) RFB4 into human V_H_ and V_L_ frameworks preselected for stability from a human antibody phage display library. The resulting humanized huRFB4 scFv (originally designated SGIII) displayed excellent antigen binding and stability properties [33]. To generate a humanized IgG1 derivative (mAb huRFB4) from the scFv the variable domain encoding genes of the humanized V_L_ chain and V_H_ chain were cloned into immunoglobulin expression vectors containing a human constant heavy *γ*1 chain and a human constant *κ* chain, respectively. The humanized antibody was produced from stably transfected Sp2/0 mouse myeloma cell lines under serum-free conditions in a hollow-fiber culture system and purified from culture supernatants to homogeneity by protein A chromatography. The purified huRFB4 IgG demonstrated specific binding to the human CD22-positive B-cell lines Daudi, Raji, and Ramos, but no binding to the human CD22-negative T-cell line Jurkat (data not shown). Flow cytometric affinity measurements confirmed that huRFB4 IgG retained the same high apparent affinity of 0.27 ± 0.02 nM as its murine ancestor mAb RFB4 (Figure 1). Bivalent binding of the humanized IgG increased the apparent binding affinity by 36-fold when compared to the parental monovalent scFv huRFB4 [33].

### 3.2. Immunoconjugation of huRFB4 IgG and ONC

**Figure 2:**
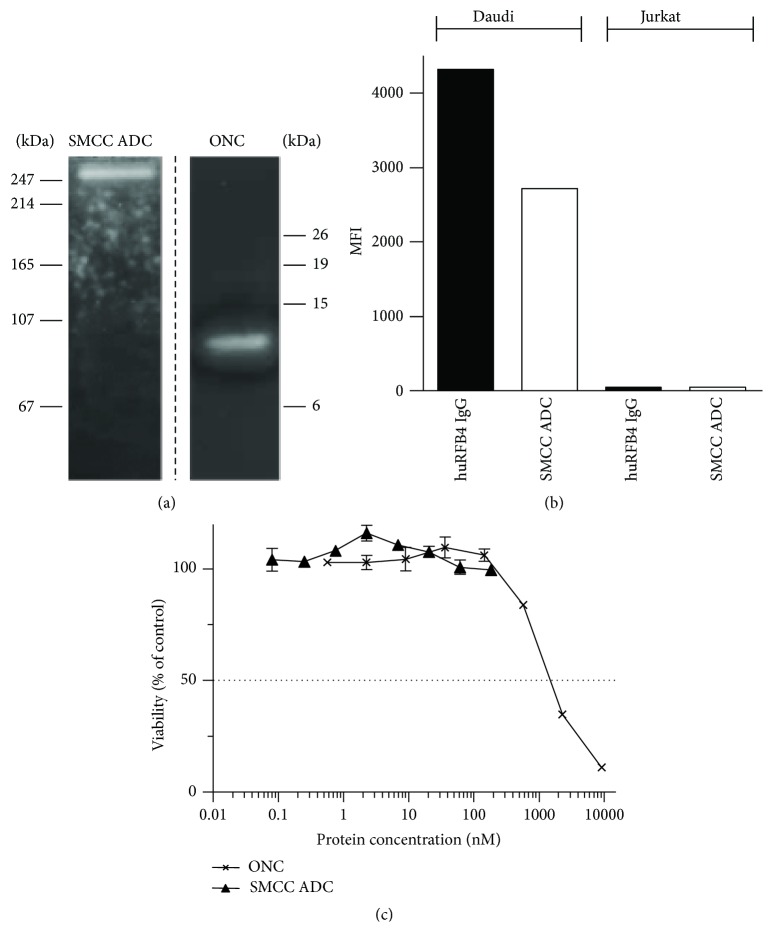
*In vitro* characterization of SMCC-based immuno-RNase ADCs. (a)* In situ* RNase activity of the immuno-RNase ADC (1.2 *μ*g) and ONC alone (2.0 *μ*g) was analyzed by zymogram gel electrophoresis on 12% SDS polyacrylamide gels containing poly-rU (0.3 mg/mL) as RNase substrate. Migration distances of molecular weight markers are indicated (kDa). (b) Binding activity of huRFB4 IgG (5 nM) and the immuno-RNase ADC (5 nM) to CD22-positive Daudi cells and CD22-negative Jurkat cells is shown as median fluorescence intensity (MFI). (c) Cytotoxicity of the immuno-RNase ADC and ONC alone towards CD22-positive Daudi cells* in vitro* was determined by cell viability assay. Results are expressed relative to buffer-treated control cells. Data depict the mean value ± SE from one representative experiment performed in triplicate.

For conjugating ONC to huRFB4 IgG we first employed the membrane permeable crosslinker succinimidyl 4-(*N*-maleimidomethyl) cyclohexane-1-carboxylate (SMCC). ONC contains four disulfide bonds of which C30/C75 is solvent exposed. We therefore reduced ONC under mild conditions and simultaneously modified the *ε*-amino groups of IgG-lysine residues via the N-hydroxysuccinimide (NHS) ester reactive group of SMCC. The SMCC-modified IgG was reacted with free accessible sulfhydryl groups of des(30-75)-ONC via the maleimide group of SMCC, generating nonreducible thioether bonds. This conjugation approach yielded 41% nonconjugated IgG (150 kDa) and 22% multimeric immuno-RNase conjugates (300 kDa) (data not shown). Although reductive unfolding of ONC into a single stable intermediate des(30-75)-ONC has been described [34, 36], reduction of the 30-75 disulfide bond in ONC for chemical conjugation has not yet been investigated. To assess if this site of conjugation interferes with the ribonucleolytic activity of ONC the 300 kDa immunoconjugates were subjected to zymography. Although multimeric immunoconjugates were enzymatically active (Figure 2(a)) and retained specific antigen binding (Figure 2(b)) they were not cytotoxic (Figure 2(c)).

**Figure 3:**
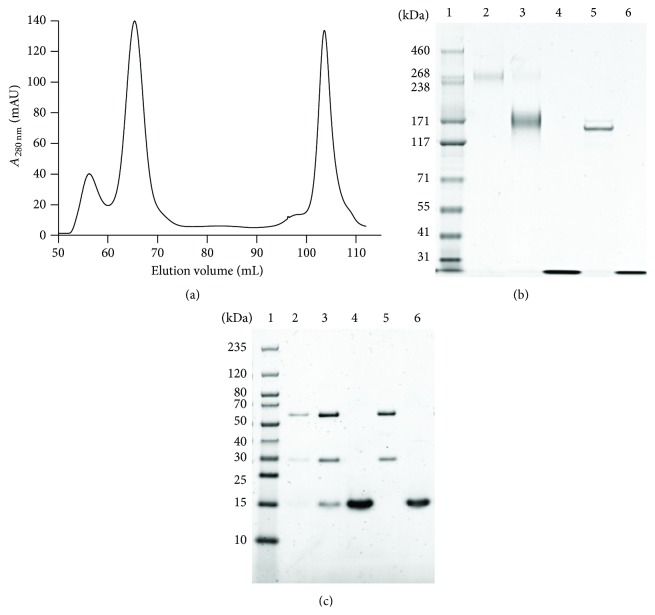
Purification of monomeric SPDP-based ADCs. (a) Size exclusion chromatography of the SPDP-based immunoconjugate preparation on a HiLoad 16/60 Superdex 200 pg column. Eluted column fractions were analyzed by SDS-PAGE under nonreducing (b) and reducing (c) conditions. Lane 1: molecular weight marker; lanes 2–4: column fractions eluting at 58.6 mL, 67.9 mL, and 106.4 mL, respectively; lane 5: huRFB4 IgG (1 *μ*g); and lane 6: ONC (1 *μ*g).

Therefore we next used the cleavable* N*-succinimidyl 3-(2-pyridyldithio) propionate (SPDP) crosslinker reacting with amino groups of ONC. Lysine residues of the huRFB4 IgG were modified by Traut's Reagent (2-iminothiolane, 2-IT) to provide an additional sulfhydryl group for subsequent immunoconjugation. Preparative size exclusion chromatography (SEC) of the SPDP-immunoconjugate preparations yielded three distinct peaks (Figure 3(a)) corresponding to 17.3% ADC multimers (58.6 mL), 70.2% immuno-RNase ADCs (67.9 mL), and 12.5% pyridyldithiol-activated ONC excess (106.4 mL) as confirmed by SDS-PAGE under nonreducing and reducing conditions (Figures 3(b) and 3(c)). Multimerization (300 kDa ADCs) was most likely attributed to small amounts of ONC molecules carrying two pyridyldithiol reactive groups, resulting in the cross-linking of two 2-IT-modified IgG molecules. Sizes of immuno-RNase ADCs were between 162 and 186 kDa (Figure 3(b)), corresponding to ONC to antibody ratios (OARs) between 1 : 1 and 3 : 1.

**Figure 4:**
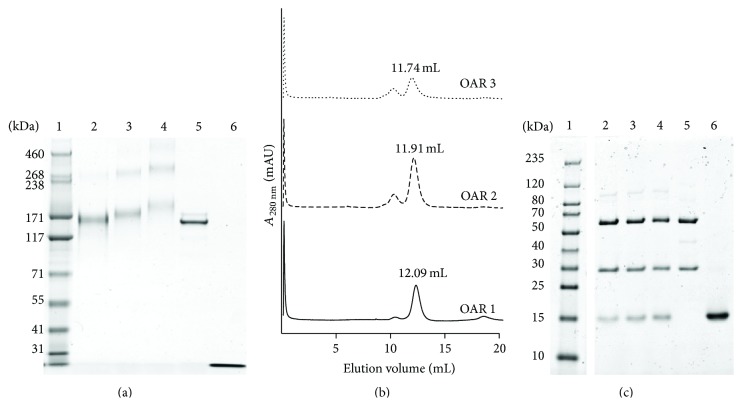
Purification of SPDP-based ADCs with distinct molar DAR. Ion exchange chromatography yielded immuno-RNases with distinct OARs as confirmed by SDS-PAGE under nonreducing (a) and reducing (c) conditions. Lane 1: molecular weight marker; lanes 2–4: ADCs with OARs of 1 : 1, 2 : 1, and 3 : 1 (1 *μ*g each); lane 5: huRFB4 IgG (1 *μ*g); lane 6: ONC (1 *μ*g). (b) Size exclusion chromatography of IEX-purified immuno-RNases was performed on a Superdex 200 10/300 GL column.

The small differences of the molecular weights of immuno-RNase ADCs with distinct OARs did not allow a preparative separation by size exclusion chromatography (Figure 3(a)). To separate immuno-RNase ADCs by their number of cytotoxic payloads we thus took advantage of the highly basic nature (pI > 9.5) of ONC [23] and subjected the conjugate pool eluting at 67.9 mL in SEC (Figure 3(a)) to a polishing step by cation exchange chromatography. By increasing the ionic strength of the elution buffer slowly from 140 to 300 mM NaCl we were able to separate intact mAb species with low ONC payloads (Figures 4(a)–4(c)). Immuno-RNase conjugates eluting at 142–174 mM NaCl, 194–221 mM NaCl, and 248–270 in ion exchange chromatography migrated under nonreducing conditions on SDS-PAGE with estimated sizes of 162 kDa, 174 kDa, and 186 kDa, respectively (Figure 4(a)). The average molecular weights of 162 kDa, 174 kDa, and 186 kDa were confirmed by subsequent calibrated SEC and correspond to distinct immuno-RNase ADCs with OAR of 1 : 1, 2 : 1, and 3 : 1, respectively. Minor ADC multimer contaminants with sizes > 300 kDa could be separated by SEC (Figure 4(b)). Analysis of immuno-RNase conjugates (defined amount of 1 *μ*g each) on reducing SDS-PAGE gels showed an OAR depending increase of the staining intensities of ONC protein bands (12 kDa), while staining of the protein bands of the respective IgG heavy and light chains remained unchanged (Figure 4(c)). When compared to the 162 kDa immunoconjugate measured densities of ONC bands increased 1.9-fold for the 174 kDa ADC and 2.5-fold for the 186 kDa ADC, which corresponds very well to the relative amounts of one, two, or three ONC loads per ADC of 6.2 pmol, 11.5 pmol, and 16.2 pmol, respectively.

### 3.3. *In Vitro* Characterization of SPDP-Based ADCs with Distinct Molar DAR

**Figure 5:**
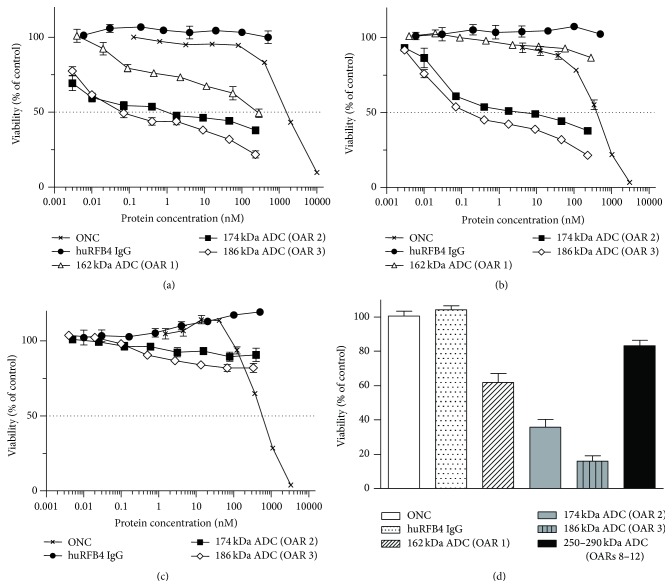
Dose- and DAR-dependent* in vitro* cytotoxicity of SPDP-based ADCs. CD22-positive Daudi (a), Nalm6 (b), and CD22-negative Jurkat (c) cells were incubated with varying concentrations of SPDP-based ADCs with distinct OARs. ONC and huRFB4 IgG were used as positive and negative control, respectively. Cell viability was determined after 72 h and is expressed relative to buffer-treated control cells. (d) Cytotoxic activities towards Daudi cells of immunoconjugates with low OAR were compared to antibody-ONC populations with higher ONC loading (OARs 8–12) at equal molar doses of 10 nM each. As immunoconjugate species with eight to twelve ONC payloads could not be further separated, molar calculations for the 250–290 kDa ADC were averaged for a payload of 10 RNases. Data depict the mean value ± SE from one representative experiment performed in triplicate.

It has been shown that conjugation procedures requiring pretreatment of ONC with reducing agents such as dithiothreitol (DTT) can diminish ribonucleolytic activity up to 60% [31]. By employing a conjugation strategy that avoids reduction of ONC we were able to fully maintain the catalytic activity of the ribonuclease in the ADC format (Table 1). As a consequence, the 162 kDa immuno-RNase ADC with an OAR of 1 : 1 showed exactly the same ribonucleolytic activity as ONC alone. The 174 kDa immuno-RNase ADC exhibited 1.9-fold increased ribonucleolytic activity according to two ONC payloads. The 186 kDa immuno-RNase ADC carrying three ONC moieties exhibited a 2.9-fold higher catalytic efficiency than ONC alone. Thus, determination of ribonucleolytic activity additionally confirmed the separation of immuno-RNases with OARs of 1 : 1, 2 : 1, and 3 : 1. Immunoconjugation had no significant impact on the biological activity of the antibody since antigen binding affinities of the immuno-RNase ADCs (*K*_*Ds*_ 0.4–0.6 nM) were comparable to the affinity of the native huRFB4 IgG (Table 1). Specific antitumor activity of the SPDP-based immunoconjugates with OARs of 1 : 1, 2 : 1, and 3 : 1 was tested on human Burkitt's lymphoma Daudi and pre-B acute lymphoblastic leukemia Nalm6 cells in comparison with CD22-negative human acute T-cell leukemia cell line Jurkat. The SPDP-based immuno-RNases meditated a dose-dependent cytotoxicity towards targeted CD22-positive tumor cells (Figures 5(a) and 5(b)) but not towards the CD22-negative leukemia cells (Figure 5(c)). Incubation of Daudi and Nalm6 cells with huRFB4 IgG alone did not result in any cytotoxicity (Figures 5(a) and 5(b)). Notably, a direct correlation between the cytotoxicity of the immuno-RNase ADCs and the number of attached cytotoxic payloads became apparent. As anticipated, cytotoxicity successively increased with the number of conjugated payloads (Table 2). In comparison with ONC alone, displaying IC_50_ values of 1.5 *μ*M on Daudi cells and 0.5 *μ*M on Nalm6 cells, targeted delivery of three ONC moieties per antibody by the most potent 186 kDa ADC resulted in enhanced cytotoxicity of 18,400-fold on Daudi cells (IC_50_ 80 pM) and 3,600-fold on Nalm6 cells (IC_50_ 140 pM) (Table 2). SPDP conjugation generated also conjugates with ONC multiplicities eluting in gel filtration chromatography on a calibrated Superdex 200 column at retention times correlating to sizes between 250 and 290 kDa and most likely representing RNase loads of eight to twelve (data not shown). Although this mixture of immunoconjugate species could not be further separated, the effect of higher ONC loading on* in vitro* potency was evaluated and compared with the immunoconjugates carrying low ONC payloads (Figure 5(d)). At equal molar doses of 10 nM, immunoconjugates with one, two, and three ONC payloads reduced the mean viability of Daudi cells by 38%, 64%, and 84%, respectively. Although cytotoxic activity correlated with drug loading levels for immunoconjugates with low OAR, immuno-RNase ADCs with higher ONC loadings displayed a significantly decreased cytotoxic activity of only 17%.

## 4. Discussion

Cytotoxic payloads currently under clinical evaluation in ADCs are antimicrotubule agents, DNA minor groove binding agents, and alkylating agents. With biological activities in the ng/kg range these compounds represent a major safety challenge both in clinical product manufacturing and in systemic application to cancer patients. The safety and therapeutic index of ADCs significantly depend not only on the reproducibility of exact attachment sites and number of attached payloads but also on the linker technology and conjugate homogeneity. Improvements in linker stability have in fact accelerated the clinical development of new generation ADCs and resulted in the recent approvals of brentuximab vedotin and ado-trastuzumab emtansine, respectively. Despite implementation of sophisticated downstream purification protocols conjugation via lysines or cysteines of the antibody results in inherent heterogeneity of the final clinical product with 0–8 drug payloads per antibody on average. To decrease heterogeneity and achieve uniform drug-loading site-specific drug attachment has recently been achieved by engineered introduction of cysteines [37] or nonnatural amino acids [38]. Employment of these technologies has resulted in ADCs with defined DAR of either two or four payloads per antibody [38–40].

In this study we employed amphibian RNase ONC as effector moiety for creating a novel protein-protein ADC to target CD22-positive leukemia and lymphoma cells. Prerequisite for the full enzymatic activity of ONC is the formation of pyroglutamate at its N-terminus through hydrogen bonding with K9 [41, 42]. It is therefore important that crosslinking of ONC to the antibody moiety must not occur via K9 of the enzyme to preserve the catalytic activity of the ribonuclease. Consequently, we explored two different conjugation strategies for analyzing the impact of the nature of the cross-linking bonds, the stoichiometry, and the efficiency of purification procedures for retaining enzymatic activity and cytotoxicity of the immuno-RNase conjugates.

Studies on the unfolding pathways of ONC have shown that ONC reductively unfolds via a single stable intermediate des(30-75)-ONC [34, 36]. It has been further shown that an ONC variant lacking the disulfide bond 30/75 mimics the unfolding intermediate des(30-75)-ONC and exhibits comparable cytotoxic properties as wild-type ONC [43]. Mild reduction of the solvent-accessible C30/C75 disulfide bond of ONC was therefore considered a feasible approach for generating active immuno-RNase ADCs. Although amine-to-sulfhydryl cross-linking with SMCC resulted in enzymatically active ADCs with sufficient binding activity, the cytotoxic activity was completely abolished. A major drawback of this conjugation approach was the uncontrolled formation of higher molecular weight immunoconjugates with impaired matrix-binding on IEX. Des(30-75)-ONC carrying two sulfhydryl groups per molecule most likely caused multimerization by cross-linking SMCC-modified IgG molecules that resulted in abolishment of cytotoxic activity.

In contrast, formation of SPDP-linked immuno-RNase ADCs via lysine residues seemed primarily to be a result of well-controllable 2-IT and SPDP cross-linking reactions, allowing for a preferential formation of immuno-RNase ADCs with favorably low DARs. High binding affinity to CD22-positive cells, well-preserved ribonucleolytic activity, and high CD22-specific cytotoxicity* in vitro* indicate no significant alterations of the molecules in critical regions, namely, the CDRs of the IgG, as well as lysine residues K9 and K31 of ONC [41].

Empirical evidence of random conjugation approaches has shown that the number of attached drugs has a significant impact on target antigen binding, systemic clearance, and antitumor efficacy of immunoconjugates [44–48]. Separation of isolated drug load species is at present only possible for dipeptide-linked ADCs, such as anti-CD30 brentuximab vedotin through hydrophobic interaction chromatography (HIC) [44, 49–51]. We have shown in the present study that IEX matrix-binding of SPDP-linked immuno-RNase ADCs not only allowed for successful purification from nonreacted IgG but also yielded homogenous ADC species with one, two, and three RNase moieties per antibody molecule. Moreover, our data revealed that the number of attached ONC payloads is crucial for achieving significant* in vitro* cytotoxicity towards CD22-positive lymphoma and leukemia cell lines. In this respect, at least two ONC payloads were required for achieving significant* in vitro* cytotoxicity and the SPDP-linked immuno-RNase ADC with a DAR of 3 : 1 was most effective. Although reasons for the significantly decreased* in vitro* potency of immunoconjugates containing eight to twelve ONC payloads per anti-CD22 mAb were not examined in closer detail our data are in line with data from other antibody-drug conjugates showing that higher drug loads increase the risk of reduced cytotoxic activity [47] most likely due to conjugational involvement of lysine residues within the complementarity determining regions (CDRs) of the IgG [48]. In addition, it has been observed in mouse xenograft models that higher drug loading levels (eight drugs per antibody) can cause an accelerated systemic clearance of ADCs which leads to a decreased therapeutic index when compared to conjugates with only four or even only two drugs per antibody [44]. Thus, similar to other optimized non-RNase-based ADCs, it is apparent that the conjugational design of chemically linked immuno-RNases should aim at a low DAR. Another option for successfully obtaining immuno-RNases with stoichiometrically defined number of ONC cargos through employment of the DOCK-AND-LOCK method has recently been reported [52]. Preferably in a head-to-head comparison advantages and disadvantages of both methodologies should be addressed in future studies.

Current clinical and preclinical development strategies for CD22-targeting ADCs and immunotoxins focus on the use of traditional cytotoxic payloads, such as calicheamicins [53], auristatins [54], maytansinoids [55], and truncated* Pseudomonas* exotoxin [21]. All of these cytotoxic agents have been associated with significant systemic toxicity either due to their off-target release through destabilization of the cross-linking bonds or because of immunogenicity, as in case of* Pseudomonas* exotoxin. Payload-dependent hemato- and hepatotoxicities, ranging from mild, reversible transaminasemia to even fatal venoocclusive disease (VOD), have been reported for the anti-CD22 calicheamicin immunoconjugate inotuzumab ozogamicin (CMC-544) used in clinical phases I–III for the treatment of patients with relapsed/refractory B-cell malignancies [53, 56–58]. Substantial postapproval safety risks attributed to VOD led to the refusal of the marketing authorization in Europe for gemtuzumab ozogamicin [5, 59, 60] in 2008 and its voluntary withdrawal from the US in 2010. Despite efforts to obtain a maximal ADC stability through introduction of noncleavable thioether bonds or intracellularly cleavable dipeptide bonds preterm payload release with potentially increased toxicity remains a major clinical problem. As with other dipeptide-linked auristatin-based immunoconjugates [61–63], preliminary results of a phase II trial of the anti-CD22-MMAE immunoconjugate pinatuzumab vedotin in combination with rituximab in patients with relapsed/refractory non-Hodgkin lymphoma reported on significant payload-related systemic toxicities including neutropenia and peripheral neuropathy [64]. Since similar toxicities were also common among patients within phase I/II trials of naked auristatin payloads [65, 66], deconjugation of the payload from the antibody with systemic release of the neurotoxic microtubule inhibitors can thus be deduced. Payload release from presumably noncleavable thioether bonds has been also demonstrated [67]: during the “thioether fragmentation reaction” the cytotoxic moieties were shown to be transferred to the unpaired C34 cysteine residue of serum albumin. Identification of albumin as a covariate affecting the pharmacokinetics of the recently FDA-approved SMCC-linked anti-HER2 trastuzumab emtansine (T-DM1) [68] might explain at least in part payload-related systemic toxicities observed in late clinical development [69]. Lately, application of self-hydrolyzing maleimide drug linkers has been shown to reduce off-target bone marrow toxicities in rats through enhanced ADC stability [70], which holds promise for future clinical development of such ADCs. While ADCs under current development represent a unique treatment option they create a series of challenges in engineering, chemistry, and safety. Alternative payloads with reversible, easily manageable systemic toxicities, yet also high antitumoral efficacy, such as ONC in the present study, represent valuable alternatives.

## 5. Conclusion

In summary, we have developed novel SPDP-linked immuno-RNase ADCs with stoichiometrically defined number of cytotoxic ONC payloads for the targeted therapy of CD22 malignancies. We have shown for the first time that the number of attached ONC payloads well correlates with the tumor-specific cytotoxicity of the ADC. Because of their highly specific toxicity towards targeted tumor cells, a fairly well-controllable conjugation and purification procedure, and expected favorable safety and immunogenicity profile we believe that further preclinical development of the 3 : 1 DAR SPDP-linked immuno-RNase ADC is warranted.

## Figures and Tables

**Table 1:** Binding affinity and ribonucleolytic activity of SPDP-based ADCs.

	OAR	Binding affinity^a^	Ribonucleolytic activity^b^
		*K*_*D*_ ± SE (nM)	*k*_cat_/*K*_*M*_ ± SE (10^3^ M^−1^s^−1^)
ONC	—	NA	14.1 ± 0.8
huRFB4 IgG	—	0.27 ± 0.02	NA
SPDP-based 162 kDa ADC	1 : 1	0.39 ± 0.03	14.1 ± 0.8
SPDP-based 174 kDa ADC	2 : 1	0.47 ± 0.03	26.5 ± 2.4
SPDP-based 186 kDa ADC	3 : 1	0.62 ± 0.03	40.2 ± 3.3

^a^Relative binding affinity (*K*_*D*_) to CD22-positive Raji cells was calculated from equilibrium-binding curves as determined by flow cytometry performed in triplicate. ^b^Ribonucleolytic activity (*k*_cat_/*K*_*M*_) was determined from at least three independent assays. Values are expressed as mean ± SE.

NA: not applicable.

**Table 2:** Dose- and DAR-dependent *in vitro* cytotoxicity of SPDP-based ADCs.

	OAR	Daudi	Nalm6	Jurkat
		IC_50_ ± SE (nM)	IC_50_ ± SE (nM)	IC_50_ ± SE (nM)
ONC	—	1,469 ± 44.50	503.2 ± 21.67	674.5 ± 99.5
huRFB4 IgG	—	>500	>500	>500
SPDP-based 162 kDa ADC	1 : 1	260.0	>284	ND
SPDP-based 174 kDa ADC	2 : 1	0.68 ± 0.30	2.20 ± 2.07	>390
SPDP-based 186 kDa ADC	3 : 1	0.08 ± 0.02	0.14 ± 0.02	>335

The concentration required to inhibit cell viability by 50% relative to buffer-treated control cells (IC_50_) was determined from semilogarithmic plots in which viability as percentage of control was plotted versus the tested protein concentration. IC_50_ values are expressed as mean ± SE and are derived from at least two independent experiments each performed in triplicate. ND: not determined.

## Abbreviations

ADC: Antibody-drug conjugate
BCP-ALL: B-cell precursor acute lymphoblastic leukemia
B-NHL: B-cell non-Hodgkin lymphoma
CDM HD: Chemically Defined Medium High Density
CDRs: Complementarity determining regions
DAR: Drug to antibody ratio
DMEM: Dulbecco's Modified Eagle's Medium
DMSO: Dimethyl sulfoxide
DTT: Dithiothreitol
EDTA: Ethylenediaminetetraacetic acid
FBS: Fetal bovine serum
FDA: Food and Drug Administration
FITC: Fluorescein isothiocyanate
G418: Geneticin disulfate
HIC: Hydrophobic interaction chromatography
IC_50_: Half maximal inhibitory concentration
IEX: Ion exchange chromatography
IgG: Immunoglobulin G
2-IT: 2-Iminothiolane
mAb: Monoclonal antibody
MES: 2-(N-Morpholino)ethanesulfonic acid
MFI: Median fluorescence intensity
MMAE: Monomethyl auristatin E
OAR: Onconase to antibody ratio
ONC: Onconase
RNase: Ribonuclease
PBS: Phosphate buffered saline
RPMI: Roswell Park Memorial Institute
scFv: Single-chain variable fragment
SDS-PAGE: Sodium dodecyl sulfate polyacrylamide gel electrophoresis
SE: Standard error
SEC: Size exclusion chromatography
SMCC: Succinimidyl 4-(*N*-maleimidomethyl) cyclohexane-1-carboxylate
SPDP: * N*-Succinimidyl 3-(2-pyridyldithio) propionate
TAA: Tumor-associated antigen
TCD: Total cumulative dose
TEMED: N,N,N,N-Tetramethylethylenediamine
Tris-HCl: Tris(hydroxymethyl)aminomethane hydrochloride
VOD: Venoocclusive disease.
